# Supplementary material for: An AI-enabled tool for quantifying overlapping red blood cell sickling dynamics in microfluidic assays
Source: Lab Chip. 2026 May 1;26(11):3366–80. doi: 10.1039/d6lc00108d (PMC13150679; doi:10.1039/d6lc00108d)
Supplement: LC-026-D6LC00108D-s001 [file LC-026-D6LC00108D-s001.pdf]

Supplementary Information (SI) for Lab on a Chip.
This journal is © The Royal Society of Chemistry 2026

Electronic Supplementary Information (ESI)

1 Effect of watershed post-processing on instance separation and cell counting across suspension densities

To evaluate the impact of watershed-based instance separation on quantitative accuracy, we compared results obtained using nnU-Net segmentation with those obtained from nnU-Net followed by watershed post-processing, using manual counting as the reference. As shown in Figure S1A–C, nnU-Net alone accurately delineates overall cell regions across all suspension densities but increasingly merges adjacent or overlapping RBCs into single connected components as cell density increases from Case 1 (A, N=62) to Case 3 (C, N=417). This merging directly propagates into counting errors, since each merged region is interpreted as a single cell instance, leading to systematic underestimation of both healthy and sickled cell counts. Applying watershed post-processing to the class-specific masks introduces marker-driven boundary propagation that separates merged regions into distinct cell instances, thereby improving agreement with manual counts across all cases. The benefit is modest at low density (Case 1, panel A) but becomes increasingly pronounced at intermediate and high densities (Case 2, panel B; Case 3, panel C), where cell overlap is prevalent. In the highest-density condition (Case 3, panel C), watershed post-processing substantially reduces discrepancies relative to manual counting at both t = 0 s (predominantly healthy cells) and t = 120 s (predominantly sickled cells). Overall, these results demonstrate that watershed post-processing is a critical step for robust instance-level counting, enabling reliable estimation of sickled-cell fractions under physiologically relevant crowding and overlap conditions.

2 Implementation details of the label-free baseline

We implemented a two-stage label-free baseline following Piansaddhayanon *et al.* [1], consisting of (i) cell detection via a region-based object detector and (ii) phenotype classification of each detected crop as shown in Figure S2. In the detection stage, we used Faster R-CNN with a ResNet-50 backbone trained in MMDetection to detect *all cells* (healthy and sickled) to ensure consistent proposal generation across phenotypes. The detector outputs, for each frame, a list of bounding boxes and detection confidence scores, which we saved as per-frame. Each entry contains $(x_1, y_1, x_2, y_2, score)$ in image coordinates. In the classification stage, we applied a ConvNext classifier [2] initialized from ImageNet-pretrained weights to assign each detected box to `Healthy (H)` or `Sickled (S)`. The backbone was initialized with ImageNet-pretrained weights [3] and optimized using SGD. For classification, we used a ConvNeXt-B classifier [2] initialized with ImageNet-pretrained weights to assign each detected cell to Healthy or Sickled. The classifier was trained on ground-truth cell crops derived from the manual annotations using Adam optimization. To ensure a fair comparison with our segmentation-based approach, we applied the same augmentation strategy during training (random flips and rotations that preserve cellular morphology) for both the detector and classifier.

3 Practical Overlap-Rate Limit of the Proposed Method

To further examine the practical overlap-rate limit of the proposed method, we performed segmentation on a video with an extremely high overlap rate of 0.923. Figure S3 shows the segmentation results at two representative time points, $t = 0\,\mathrm{s}$ and $t = 120\,\mathrm{s}$. The first column presents the original images, which exhibit severe cell crowding and extensive overlap. The second column shows the nnU-Net predictions, where healthy and sickled RBCs are correctly identified at the semantic level, although many overlapping cells remain connected. The third column shows the results after applying the watershed-based post-processing, which further separates clustered and overlapping cells into more distinguishable individual instances, thereby improving instance-level counting. The quantitative results in Table 3 further demonstrate the robustness of the proposed framework under this extremely challenging condition. At $t = 0\,\mathrm{s}$, the manual counts were 425 healthy cells and 62 sickled cells, while the predicted counts were 401 and 73, respectively. At $t = 120\,\mathrm{s}$, the manual counts were 94 healthy cells and 411 sickled cells, compared with predicted counts of 111 and 381. Overall, the predicted results remain in close agreement with manual counting even at an overlap rate as high as 0.923, indicating that the proposed nnU-Net + watershed framework remains reliable under very dense and highly overlapping cell distributions. We did not further increase the overlap rate beyond this level. The

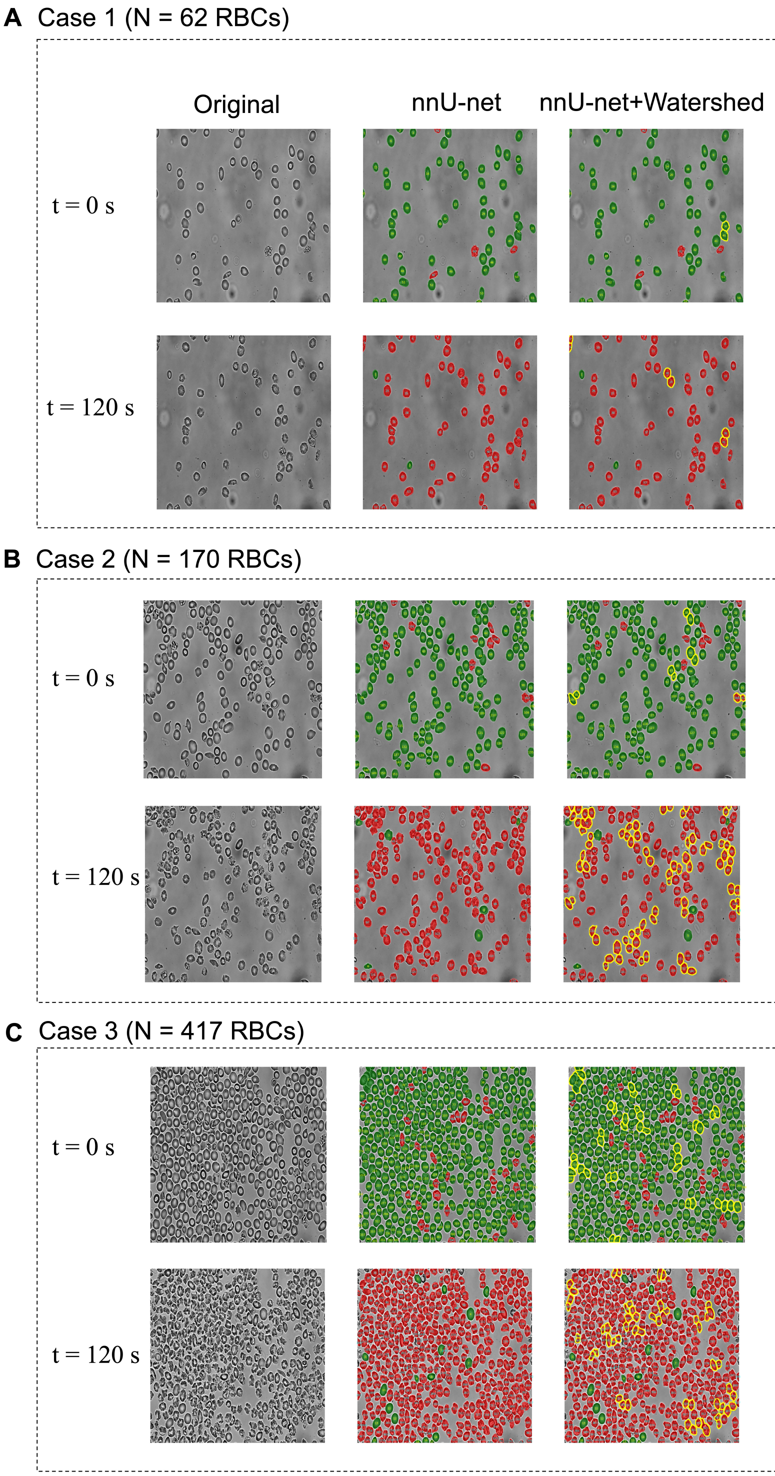

Method	#Healthy	#Sickle
**Case 1, t = 0 s**		
nnU-Net	58	4
nnU-Net + watershed	59	4
Manual count	58	4
**Case 1, t = 120 s**		
nnU-Net	2	57
nnU-Net + watershed	2	60
Manual count	3	60
**Case 2, t = 0 s**		
nnU-Net	153	11
nnU-Net + watershed	157	12
Manual count	157	12
**Case 2, t = 120 s**		
nnU-Net	6	120
nnU-Net + watershed	6	161
Manual count	5	164
**Case 3, t = 0 s**		
nnU-Net	358	32
nnU-Net + watershed	379	32
Manual count	382	35
**Case 3, t = 120 s**		
nnU-Net	18	366
nnU-Net + watershed	18	394
Manual count	19	400

Figure S1: **Effect of watershed post-processing on instance separation and cell counting across suspension densities.** Representative frames are shown for three cell population densities: Case 1 ($N = 62$), Case 2 ($N = 170$), and Case 3 ($N = 417$), at $t = 0$ s and $t = 120$ s. Columns display the original image, nnU-Net predictions without watershed post-processing (which tend to merge adjacent or overlapping cells), and nnU-Net predictions with watershed-based instance separation (which splits merged regions into individual cell instances). The accompanying tables report the corresponding healthy and sickled cell counts from each method and from manual counting. In the overlays, green indicates healthy cells, red indicates sickled cells, and yellow highlights regions where overlapping cells are successfully separated by watershed.

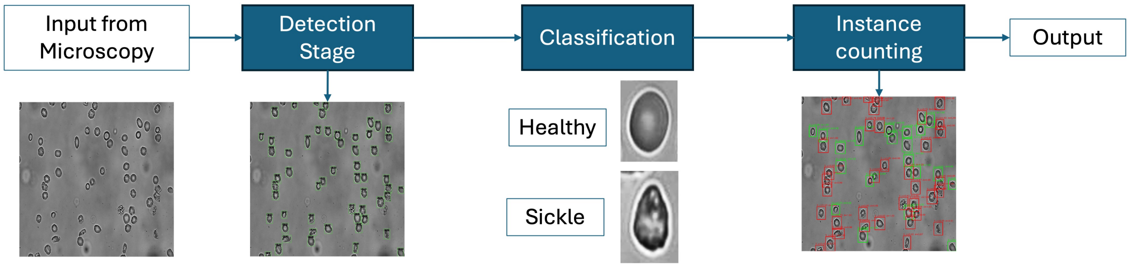

Figure S2: **Label-free baseline schematic.** A two-stage pipeline comprising a detector (Faster R-CNN with ResNet-50 backbone) that proposes cell bounding boxes, followed by a ConvNeXt-based classifier that assigns each detected crop to healthy (green) or sickled (red) classes.

reason is that, under even more severe overlap, manual counting itself becomes increasingly ambiguous and unreliable, making it difficult to establish a sufficiently accurate ground truth for quantitative validation. Therefore, the video with an overlap rate of 0.923 can be regarded as a practical upper-limit test for the current validation study.

Time	Healthy (Manual)	Healthy (Prediction)	Sickled (Manual)	Sickled (Prediction)
$t = 0\,\mathrm{s}$	425	401	62	73
$t = 120\,\mathrm{s}$	94	111	411	381

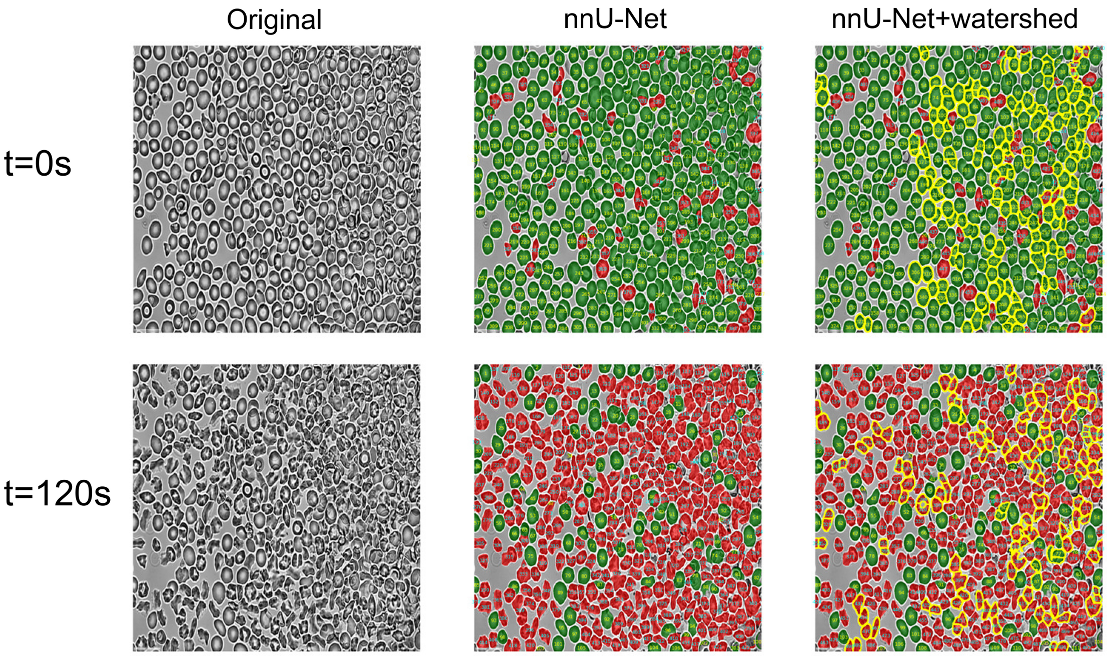

Figure S3: Segmentation results for a video with an overlap rate of 0.923, shown at two representative time points, $t = 0\,\mathrm{s}$ and $t = 120\,\mathrm{s}$. From left to right, the columns present the original images, the nnU-Net predictions, and the nnU-Net + watershed results.

References

[1] Chawan Piansaddhayanon, Chonnuttida Koracharkornradt, Napat Laosaengpha, Qingyi Tao, Praewphan Ingrungruanglert, Nipan Israsena, Ekapol Chuangsuwanich, and Sira Sriswasdi. Label-free tumor cells classification using deep learning and high-content imaging. *Scientific Data*, 10(1):570, 2023.

[2] Zhuang Liu, Hanzi Mao, Chao-Yuan Wu, Christoph Feichtenhofer, Trevor Darrell, and Saining Xie. A convnet for the 2020s. In *Proceedings of the IEEE/CVF conference on computer vision and pattern recognition*, pages 11976–11986, 2022.

[3] Jia Deng. A large-scale hierarchical image database. *Proc. of IEEE Computer Vision and Pattern Recognition, 2009*, 2009.
